# Children With Developmental Coordination Disorder Exhibit Greater Stepping Error Despite Similar Gaze Patterns and State Anxiety Levels to Their Typically Developing Peers

**DOI:** 10.3389/fnhum.2020.00303

**Published:** 2020-07-28

**Authors:** Johnny V. V. Parr, Richard J. Foster, Greg Wood, Mark A. Hollands

**Affiliations:** ^1^Department of Sport and Exercise Sciences, Research Centre for Musculoskeletal Science and Sports Medicine, Manchester Metropolitan University, Manchester, United Kingdom; ^2^Research to Improve Stair Climbing Safety, Faculty of Science, School of Sport and Exercise Sciences, Liverpool John Moores University, Liverpool, United Kingdom

**Keywords:** developmental coordination disorder, fall-risk, gaze, kinematics, anxiety

## Abstract

This study examined stepping accuracy, gaze behavior, and state-anxiety in children with (*N* = 21, age *M* = 10.81, *SD* = 1.89) and without (*N* = 18, age *M* = 11.39, *SD* = 2.06) developmental coordination disorder (DCD) during an adaptive locomotion task. Participants walked at a self-selected pace along a pathway, placing their foot into a raised rectangular floor-based target box followed by either no obstacles, one obstacle, or two obstacles. Stepping kinematics and accuracy were determined using three-dimensional motion capture, whilst gaze was determined using mobile eye-tracking equipment. The children with DCD displayed greater foot placement error and variability when placing their foot within the target box and were more likely to make contact with its edges than their typically developing (TD) peers. The DCD group also displayed greater variability in the length and width of their steps in the approach to the target box. No differences were observed between groups in any of the gaze variables measured, in mediolateral velocity of the center of mass during the swing phase into the target box, or in the levels of self-reported state-anxiety experienced prior to facing each task. We therefore provide the first quantifiable evidence that deficits to foot placement accuracy and precision may be partially responsible for the increased incidence of trips and falls in DCD, and that these deficits are likely to occur independently from gaze behavior and state-anxiety.

## Introduction

Developmental Coordination Disorder (DCD), also known as dyspraxia, affects around 5% of children and is characterized by difficulties in general motor skill learning and execution, which are independent of intellectual problems, visual impairments, and physical or diagnosed neurological disorders (American Psychiatric Association, 2013). The movements of children with DCD are often described as awkward or clumsy and affect the ability to perform activities of daily living (ADLs). For example, children with DCD struggle walking around the environment safely (Van der Linde et al., 2015); an often overlooked skill that requires a complex interaction between the central nervous system, musculoskeletal system, sensory inputs from the visual, proprioceptive and vestibular systems, and environmental cues (Rossignol, 1996). Indeed, children with DCD appear to use shorter steps and a bent-forward posture to optimize safety when walking on a treadmill (Deconinck et al., 2006a) and display a reduced ability to control their momentum when crossing obstacles (Deconinck et al., 2010). Children with DCD also trip, fall, and bump into obstacles more frequently than their typically developing (TD) peers (Fox and Lent, 1996; Cleaton et al., 2020) which can negatively impact everyday life and the willingness to engage in sports and social activities (Kirby et al., 2011). Problems with walking can extend into adulthood, as exemplified by a recent study that showed adults with DCD reported falling more than 10 times over a 6-month period and tripping between one and five times per week (Scott-Roberts and Purcell, 2018).

Although laboratory studies have demonstrated that stability of gait is lower in children with DCD (Gentle et al., 2016; Speedtsberg et al., 2018) and that individuals with DCD fail to show key anticipatory adjustments when negotiating obstacles suddenly appearing in their walking path (Wilmut and Barnett, 2017), the mechanisms underpinning these differences have not been fully elucidated. Problems with internal (forward) modeling, balance control, rhythmic coordination, executive function, and aspects of sensoriperceptual function have been implicated as possible mechanisms of motor deficits in individuals with DCD (Wilson et al., 2013) but there is no direct evidence that these mechanisms can explain DCD-related changes in gait and posture. Therefore, there is a clear need for further exploration of the mechanisms underpinning walking problems of individuals with DCD so that effective interventions can be designed and implemented.

One potential mechanism of walking difficulties in DCD is the coupling between the visual and locomotor system. When navigating complex environments vision is critical for the acquisition of necessary information to guide safe stepping behavior. For example, when faced with stepping over a future obstacle, individuals typically look several steps ahead, fixating the obstacle and other task-relevant areas to plan future foot placement (Patla and Vickers, 2003; Marigold and Patla, 2007; Matthis et al., 2018). Additionally, when stepping onto a target, individuals tend to transfer their gaze toward the target prior to step initiation and maintain this fixation until around the time the step is completed (Hollands et al., 1995; Hollands and Marple-Horvat, 2001). It has been suggested that eye and stepping movements are programmed simultaneously as part of a coordinated eye-stepping movement (Hollands and Marple-Horvat, 2001), and that problems making accurate eye movements may lead to problems making accurate stepping movements (Hollands et al., 2017). It is, therefore, noteworthy that the oculomotor control of children with DCD differs from that of their TD peers. For example, children with DCD are less accurate during saccadic transitions to spatial targets (Katschmarsky et al., 2001) and struggle when faced with visually tracking a moving target (Robert et al., 2014; Sumner et al., 2018). Children with DCD also tend not to use predictive information to guide the planning of subsequent movements (Langaas et al., 1998; Wilmut and Wann, 2008; Ferguson et al., 2015), instead showing a preference to rely on visually guided online control (Smits-Engelsman et al., 2003; Wilmut et al., 2006; Debrabant et al., 2013) which has been shown to impair their ability to visually track and catch a ball (Miles et al., 2015). Importantly, these differences have recently been shown to persist in the context of walking (Warlop et al., 2020). Specifically, when faced with navigating sequential stepping targets, young adults with DCD walk slower and direct their gaze to the more proximal and immediate stepping targets compared to their TD peers. Difficulties using predictive control may therefore alter what is perceived to be the most task-relevant sources of visual information to guide action (Land and Lee, 1994; Land and Hayhoe, 2001) and encourage individuals with DCD to utilize slower (and online) sources of sensory feedback (Adams et al., 2014). Consequently, the extent to which vision is used to sufficiently identify and plan for subsequent stepping constraints may be limited. However, it is currently unknown whether these visuomotor deficits are also observed in children with DCD, and whether they contribute to decreased stepping accuracy and associated increased risk of falls.

Another potential mechanism for the movement problems in DCD, that has been hitherto unexplored, is the link between stepping accuracy and mental health issues, such as anxiety (Caçola, 2016). There is growing evidence that individuals with DCD have elevated levels of anxiety compared to their TD peers (Mancini et al., 2016, 2019; Omer et al., 2019), and that increased anxiety pertaining to mobility results in some adults with DCD exerting conscious effort to maintain balance and avoid tripping and falling (Scott-Roberts and Purcell, 2018). Whilst there is little known about any link between anxiety and fall risk in DCD, fear of falling is a known risk factor for falls in older adults and certain patient populations (Lord et al., 1993; Cumming and Klineberg, 1994; Delbaere et al., 2010) and can lead to changes to walking behavior that paradoxically increases the risk of tripping and falling (Young and Hollands, 2010, 2012b; Young et al., 2012; Young and Williams, 2015). For example, when approaching a stepping target followed by a series of obstacles, older adults with a high-risk of falling show a reduced tendency for feedforward and proactive visual search behaviors compared to their low-risk counterparts (Young et al., 2012). That is, they are more likely to only fixate the most immediate stepping constraints at the expense of sufficiently fixating the more distal and subsequent stepping constraints. This results in high-risk older adults, who also report heightened state-anxiety, sometimes looking away too early from the target box they are stepping onto which can result in inaccurate foot placement (Chapman and Hollands, 2006, 2007, 2010; Young and Hollands, 2012a; Young et al., 2012; Young and Williams, 2015). These findings therefore suggest that the increased likelihood of trips and falls in older adults are due, in part, to not looking in the right places at the right times; behavior shown to be directly linked to the effects of anxiety/fear of falling on attentional control processes (Young and Hollands, 2012b; Young and Williams, 2015; Ellmers and Young, 2019). Though it is currently unclear whether falls in older adults and children with DCD share common etiologies, the influence of anxiety on visuomotor control is a mechanism that may explain problems with effective gait in DCD populations.

The aim of the current experiment was to provide the first detailed account of the visuomotor control of stepping in children with and without DCD and to determine the extent to which deficits in stepping accuracy may be explained by anxiety and gaze behavior. Building upon recent insights to gaze behaviors during precision stepping in adults with DCD (Warlop et al., 2020), we report how children with and without DCD use gaze to preview a varying number of stepping constraints prior to precise foot placement within a floor-based target – providing the first quantification of foot placement error in children with and without DCD. We hypothesized that compared to their TD peers, children with DCD would display (1) greater foot placement error, (2) altered visual sampling during the approach to, and stepping into, our floor-based target, and (3) heightened levels of state-anxiety.

## Materials and Methods

### Participants

Forty-seven participants aged between 8 and 15 years of age participated in the study, of which 28 were initially recruited for our DCD group. Participants in the DCD group were recruited using social media and from local DCD support groups, whilst participants in the TD group were recruited from the children of student and staff members of Liverpool John Moores University. The children in the DCD group satisfied the Diagnostic and Statistical Manual of Mental Disorders (DSM-5) criteria (American Psychiatric Association, 2013). For example, the Developmental Coordination Disorder Questionnaire (DCDQ; Wilson et al., 2009) was completed by parents prior to testing to confirm that movement difficulties significantly interfered with their child’s activities of daily living. Parents also confirmed that their child did not suffer from any general medical condition known to affect sensorimotor function (e.g., cerebral palsy, hemiplegia, or muscular dystrophy) and had no diagnosis of learning difficulties. Finally, participants in the DCD group were required to score below the 15th percentile on the test component of the Movement Assessment Battery for Children-2 (MABC-2; Henderson et al., 2007) carried out as part of the testing phase. This resulted in five participants’ data being excluded from our analyses (min = 25th percentile). A further two participants were also excluded from the DCD group due to poor adherence to task instructions. Participants in the TD group were required to score above the 15th percentile, which resulted in the exclusion of one participant’s data. This resulted in a net total of 21 participants in our DCD group (male = 12, female = 9) and 18 participants in our TD group (male = 10, female = 8). All participants were right footed. Parents also completed the Attentional Deficit/Hyperactivity Disorder (ADHD) Rating Scale —VI prior to testing (DuPaul et al., 1998) due to its high comorbidity with DCD (about 50% co-occurrence; American Psychiatric Association, 2013). None of the included children scored above the 98th percentile for inattention or hyperactivity, which is recommended to be the minimum cut-off used as an indication of ADHD in research (DuPaul et al., 1998). Ethical approval was granted by the Liverpool John Moores University Ethics Committee.

### Kinematics

A 12 infra-red camera motion capture system (Qualisys, Gothenburg, Sweden) collected whole-body kinematic data at 80 Hz, with a total of 38 reflective markers placed on the feet, lower legs, thighs, pelvis, torso and head according to the conventional Plug-in Gait marker set. This included several additional markers to optimize segment tracking, one of which was placed on the “foot center” to guide each child’s stepping behavior (see below). Finally, a triangular cluster of three reflective markers (14 mm diameter) were placed on each shoe over the forefront to track virtual landmarks created by a digitizing wand (C-Motion, Germantown, MD, United States) at the anterior-inferior (toe-tip) and posterior-inferior (heel-tip) point of each shoe. Marker trajectories were labeled and gap-filled using Qualisys Track Manager (QTM) before being exported as.c3d files to enable model application in Visual3D (C-Motion, Germantown, MD, United States). Finally, data were exported and analyzed in MATLAB (MathWorks, United States). All trajectories were smoothed using a bi-pass second order Butterworth low-pass digital filter with a 6 Hz cut-off.

### Eye Tracker

Eye movements were recorded using a Pupil Labs binocular eye-tracking headset (Kassner et al., 2014) that featured two pupil cameras that recorded pupil movements at 60 Hz, and a scene camera to record the world view at 30 Hz. Prior to the task, children completed a 5-point screen marker calibration that was re-run every five trials or when the calibration accuracy had visibly been lost. If the child failed calibration after multiple attempts, or persistently lost calibration due to excessive movement of the eye-tracker, the task was run without the eye-tracker and their gaze data excluded. Participants were also only included in gaze analyses if they presented two or more usable eye-tracking trials per condition. In total, this resulted in the gaze data from 5 DCD children being excluded from the present study (age = 10.00 ± 0.71, Mabc-2 = 2.42 ± 3.75). Of the participants included in gaze analyses, an average of 1.68 trials (11.17%) in total (15 trials) were rejected from analyses (SD = 2.26, 15.24%). The characteristics of the DCD children included in the gaze analyses is presented in Table 1. The capture onset of the motion capture system provided a light emitting diode (LED) response that enabled synchronization between the eye tracker and the motion-capture system by identifying the frame in which this response was first seen.

**TABLE 1:** Characteristics (mean ± SD) of all participants in the DCD and TD groups, and the characteristics of the subset of DCD participants whose gaze data were included in analyses.

	**DCD**	**TD**	**DCD (gaze analyses)**
Male (*n*)	12	10	10
Female (*n*)	9	8	6
Age (years)	10.811.89	11.392.06	11.062.08
Height (cm)	152.2510.99	149.0813.06	153.5011.11
Weight (kg)	49.6314.05	42.2114.69	51.2014.41
Mabc-2 (%)	1.602.51	52.9431.03	1.342.08

### Protocol

Data collection took place in a single session lasting approximately 2 h. Once fitted for kinematic and eye-tracking data collection, each child was permitted up to 2 min to walk freely around the lab to familiarize walking at their natural walking speed whilst wearing the testing equipment. Once their natural walking speed was agreed upon (confirmed by parent), the lab was marked out ready for the testing protocol. Baseline levels of state-anxiety were then measured using a child-friendly “fear thermometer”^1^, which encompasses a 10-point “smiley-face” Likert scale ranging from 1 (low levels of anxiety) to 10 (high levels of anxiety). Specifically, each child was sat down on a chair and given a brief introduction to the thermometer. They were then asked how worried or anxious they currently felt about being in the laboratories and wearing our equipment. These simple scales have previously been validated against larger and more complex state-anxiety inventories (Houtman and Bakker, 1989).

**FIGURE 1:**
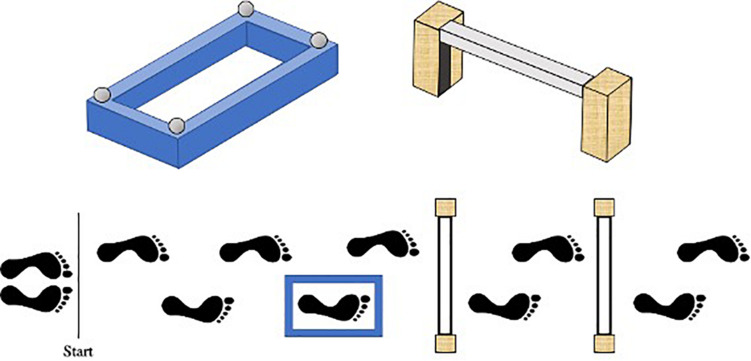
Birds-eye schematic of our walking task. Starting with their left foot, participants had to walk along the path, step into the blue target box, and over either no obstacles, one (nearest) obstacle or two obstacles. The distance between the start line, target box, and obstacles was personalized to each child’s preferred walking speed, such that their fourth step would naturally place their right foot into the target box, and their sixth and eighth steps would place their right foot over the first and second obstacles, respectively.

The present study adopted a modified-version of the protocol previously used by Curzon-Jones and Hollands (2018) to investigate stepping safety in older adults. Specifically, participants were required to walk along a 7 m path, starting with their non-dominant foot, stepping accurately into a target box and over a varying number of obstacles until they reached the end of the course. The distance between the start-line, target box, and obstacles was personalized to each child’s natural walking speed, such that their fourth step would intuitively place their dominant foot into the target box, and their sixth and eighth steps would place their dominant foot over the first and second obstacles, respectively (see Figure 1). To achieve this, each child walked along the entire pathway stepping onto a small sponge placed approximately where the target box center would later be located. The starting position was then adjusted until the above criteria were met.

The target box was a raised blue rectangular sponge outline with edges that were 5 cm high and 4 cm wide. Bespoke target boxes were created for each participant, ensuring that the length of the inside stepping area was 8cm plus the length at the longest part of the participant’s right shoe, and the width was 8cm plus the width at the widest point of the participant’s right shoe. The obstacles were formed using two 30 cm × 10 cm × 5 cm (height × depth × width) stabilizing wooden blocks positioned either side of a 4 cm × 4 cm × 65 cm polystyrene rectangular block. To also ensure the obstacles presented the same stepping constraint for each participant, the polystyrene block was attached to each stabilizing block using Velcro so it could be positioned at a height equating to 12% of body height. This height was chosen to closely match the constraints previously shown to induce fall-related anxiety in older adults (Curzon-Jones and Hollands, 2018).

Participants were informed that their goal was to reach the end of the course without knocking over the target box and/or the obstacles. Participants were also informed that they should step into the target box “as accurately and centrally as possible” – doing their best to minimize the distance between the additional “foot center” marker and what they perceived to be the center of the box. Three task difficulties were used: (1) no obstacles following the target box (Target only), (2) one (near) obstacle following the target box (One obstacle), (3) two (near and far) obstacles following the target box (Two obstacles). Participants completed five successive trials of each difficulty (one block). The order of each block was randomized (total 15 trials). Participants started each trial with their eyes closed, and after ten seconds, were verbally cued to “open” their eyes and initiate the trial. Starting each trial in this manner enabled route-previewing to be better standardized across participants and provide a point from which gaze data could be recorded. Immediately prior to each block of 5 trials, participants were again asked to report their levels of state-anxiety to determine how task difficulty influenced anxiety. Specifically, each child was asked how worried or anxious they currently felt about performing the upcoming set of trials.

### Data Analysis

#### Foot Placement Variables

Foot placement error within the target box was determined as the relative distance between the foot center and the target center when the foot was placed inside the target. Foot center was calculated as the mid-point between the toe-tip and heel-tip. Target center was calculated as the mean of the four reflective marker (x, y) coordinates positioned on each corner of the target box. Both absolute error, constant error and variable error were calculated in the anteroposterior and mediolateral directions separately. Absolute error was defined as the mean scalar foot position distance (regardless of position) relative to the target center, reflecting foot placement accuracy. Constant error was defined as the mean vector foot position displacement (±) relative to the target, reflecting foot placement bias. Variable error was defined as the variability (one standard deviation) of the constant foot placement error across trial repetitions, reflecting precision of foot placement (Reynolds and Day, 2005; Chapman et al., 2012). Unlike absolute error, constant error captures directional foot placement bias as the mean vector foot displacement (±) relative to the target and is therefore better placed to measure variability. Positive values for anteroposterior and mediolateral constant error indicate the foot was positioned anterior and lateral of the target center, respectively. Finally, the experimenter manually recorded the total number of trials (out of 15 trials) that each participant accidentally contacted the target box. The lightweight design of the target box meant even a slight touch on its edges would result in a distortion to its rectangular shape and often knock it over. This allowed the experimenter to easily determine when the box had been contacted so it could be reset for the following trial.

#### Stepping Kinematics and Approach Speed

Heel-strike and toe-off gait events were determined using the local maxima and local minima of the heel and toe referenced to the pelvis segment, respectively (Zeni et al., 2008). Using these gait events, spatial step kinematics were calculated based on the position of the foot center (mid-point between the toe-tip and heel-tip). Step length was defined as the antero-posterior distance between the left and right foot centers at each heel-strike. Step width was defined as the medio-lateral distance between the left and right foot centers at each heel-strike. As these measures are highly dependent on body morphology, we chose to measure the variability (one standard deviation) in the length and width of the steps up to and including the final step into the target box, which can give insights to the ability to produce consistent movement patterns (Rosengren et al., 2009). Approach velocity was calculated as the mean horizontal velocity of the anterior trunk marker, from the first heel strike to the instant of touch-down within the target box. Finally, we examined balance control by measuring the maximal mediolateral velocity of the center of mass (CoM) during the swing phase of the targeting step into the box (Deconinck et al., 2010). Variability of this measure was also calculated as one standard deviation across each block of trials.

#### Gaze Variables

Gaze fixations were defined as a gaze stabilization on a location in the environment for three frames or longer (corresponding to ∼90 ms). Fixations were classified as being spatially located on one of three primary areas of interest: (1) immediate walkway (walkway preceding target box); (2) target box; (3) distal walkway (the sum of all fixations directed toward the path and/or stepping constraints following the target box). We chose to classify distal fixations as a single area of interest given their low summed-frequency, and to allow comparisons between the three task difficulties. These areas of interest were used to determine the duration spent fixating each location prior to stepping in the target box. Fixation durations were also normalized to individual trial length by presenting data as the percentage of time spent fixating each area of interest from the point when participants opened their eyes following the “open” cue, until the time when they stepped into the target box. We also measured the timing of the final gaze transfer toward the target box and the final gaze transfer away from the target box relative to foot contact within it, with a negative value denoting an early transfer of gaze. Other gaze variables included mean fixation duration, fixation rate (number of fixations per second), and number of gaze transfer between areas of interest.

### Statistical Analyses

Kinematic and gaze variables were primarily analyzed using two-way mixed design repeated measures ANOVAs, with between-subject effects of group (x2; DCD; TD), within-subject effects of task difficulty (x3; Target only; One obstacle; Two obstacles), and interaction between terms. Significant effects were probed by polynomial trend analyses, and *post hoc* analyses were performed using pairwise comparisons with Sidak-corrections to account for the multiple comparison problem (Blakesley et al., 2009). ANOVA effect sizes were reported using partial eta squared (η*_*p*_*^2^), common indicative thresholds for which are small (0.01), medium (0.06) and large (0.14; Field, 2013). The results of univariate tests are reported, with the Huynh-Feldt correction procedure applied for analyses that violated the sphericity of variance. For step length variability, a natural-log transformation was applied to achieve a normal distribution. Where a normal distribution could not be achieved, within-participant effects were analyzed using Friedman’s ANOVA with Bonferroni corrected Wilcoxon-signed rank tests used for *post hoc* analyses. Conversely, between-participant effects were analyzed using Mann-Whitney U tests. Non-parametric effect sizes were reported as *r* = *Z/*$\sqrt{N}$, for which common thresholds are small (0.1), medium (0.3) and large (0.5; Rosenthal, 1986). All statistical analyses were performed using IBM SPSS statistics (version 26) with an alpha level of ≤ 0.05.

## Results

### Foot Placement Variables

#### Stepping Accuracy and Precision

**FIGURE 2:**
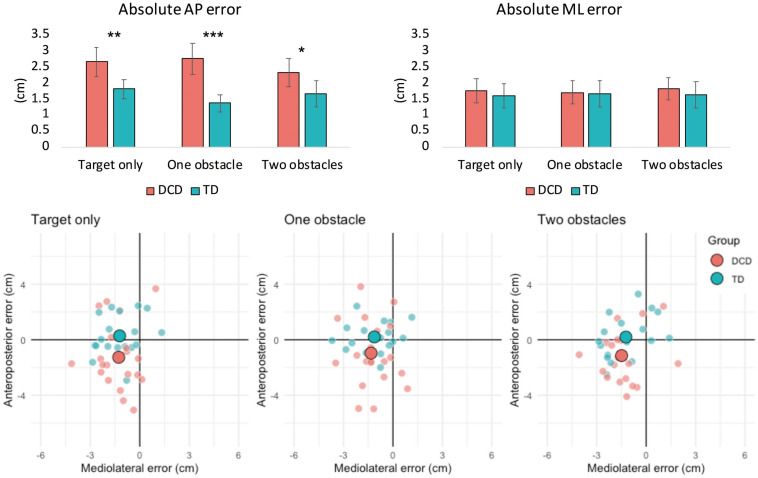
Bar charts (top) representing mean (± 95% CI) absolute foot placement error in both the anteroposterior (left) and mediolateral (right) directions of movement. Asterisks signify between group differences at the < 0.05 (*), 0.01 (**), and 0.00 (***) levels. Constant foot placement error (bottom) for the DCD and TD groups for the target only, one obstacle and two obstacle task conditions. The large data points represent the group means, whilst the smaller data points represent the mean values of individual participants. Negative values on the horizontal and/or vertical axes indicate that the foot was positioned medial and/or posterior of the target center, respectively.

There was a significant main effect of Group on absolute AP error, *F*(1, 37) = 21.063, *p* < 0.001, η*_*p*_*^2^ = 0.363, and constant AP error, *F*(1, 37) = 7.020, *p* = 0.012, η*_*p*_*^2^ = 0.159. Children with DCD had greater absolute AP error (*M* = 2.6 cm) compared to TD children (*M* = 1.6 cm) and tended to undershoot their foot placement (*M* = −1.1 cm) compared to TD children (*M* = 0.2 cm). A significant main effect of Group was also observed for AP, *F*(1, 37) = 9.932, *p* = 0.003, η*_*p*_*^2^ = 0.212, and ML variable error, *F*(1, 37) = 10.011, *p* = 0.003, η*_*p*_*^2^ = 0.213. Children with DCD exhibited greater AP (*M* = 2.1 cm) and ML (*M* = 1.3 cm) variable error compared to TD children (*M* = 1.5 cm and M = 0.9 cm, respectively). There was also no main effect of Difficulty, or interaction between Difficulty and Group, for all foot placement variables (Figure 2).

#### Total Box Contacts

Results from a Mann–Whitney *U*-test showed a significant difference between groups, *U* = 118, *z* = −2.076, *p* = 0.038, *r* = -0.3329, with more box contacts observed in the DCD group (*M* = 1.82 ± 1.41) compared to the TD group (*M* = 1.00 ± 1.24).

### Stepping Kinematics and Approach Speed

#### Approach Speed

The main effect of difficulty failed to reach significance, *F*(2, 74) = 2.968, *p* = 0.058, η*_*p*_*^2^ = 0.074, but was significantly described by a linear polynomial trend (*p* = 0.022, η*_*p*_*^2^ = 0.134) with fastest approach speeds observed when faced with the target only (*M* = 0.995 m/s) and slowest approach speeds observed when faced with two obstacles (*M* = 0.969 m/s). There was no main effect of Group, *F*(1, 37) = 3.273, *p* = 0.079, η*_*p*_*^2^ = 0.081, and no Group × Difficulty interaction, *F*(2, 74) = 0.174, *p* = 0.841, η*_*p*_*^2^ = 0.005.

#### Stepping Variability

Results showed a significant main effect of Group for step length variability, *F*(1, 37) = 6.423, *p* = 0.016, η*_*p*_*^2^ = 0.148, with greater variability observed in the DCD group (*M* = 10.6 cm) compared to the TD group (*M* = 6.7 cm). There was also a main effect of Group for step width variability, *F*(1, 35) = 4.958, *p* = 0.032, η*_*p*_*^2^ = 0.124, with greater variability again observed in the DCD group (*M* = 5.0cm) compared to the TD group (*M* = 3.8 cm). There was no main effect of Difficulty and no Group x Difficulty interaction for either step length or step width variability.

#### Mediolateral CoM Velocity

There was no main effect of Group, *F*(1, 37) = 0.128, *p* = 0.722, η*_*p*_*^2^ = 0.003, no main effect of Difficulty, *F*(2, 74) = 0.228, *p* = 0.796, η*_*p*_*^2^ = 0.006, and no Group x Difficulty interaction, *F*(2, 74) = 1.00, *p* = 0.373, η*_*p*_*^2^ = 0.026, in the maximal mediolateral CoM velocity during the swing phase into the box. There was also no effect of Group, *F*(1, 37) = 1.762, *p* = 0.193, η*_*p*_*^2^ = 0.045, no effect of Difficulty, *F*(2, 74) = 0.137, *p* = 0.872, η*_*p*_*^2^ = 0.004, and no Group × Difficulty interaction, *F*(2, 74) = 1.914, *p* = 0.155, η*_*p*_*^2^ = 0.049, in the inter-trial variability (1 SD) of maximal CoM mediolateral velocity.

### Gaze Behavior

Gaze fixations to task related areas of interest accounted for an average of 75.0, 73.7 and 73.5% of the total time taken to step into the target box for the Target-only, One obstacle, and Two obstacle conditions, respectively. There were no significant differences between groups for fixation duration, fixation rate, number of gaze transfers between AOI’s, the total time spent fixating each AOI, or the onset of the final gaze shift toward the target prior to heel contact. These data are presented in Table 2.

**TABLE 2:** Mean (± SD) values of gaze variables and state anxiety for both the DCD and TD groups for each of the three task difficulties.

	**DCD**			**TD**		
	**Target only**	**1 obstacle**	**2 obstacles**	**Target only**	**1 obstacle**	**2 obstacles**
Immediate walkway (%)	10.6314.00	11.7513.38	9.4412.23	11.1210.22	10.4410.73	11.7213.22
Target box (%)	61.0014.61	53.6915.13	55.5615.06	59.0017.12	56.6114.02	53.2816.51
Distal (%)	3.563.79	8.947.34	8.068.15	5.136.77	6.566.92	8.289.10
Immediate walkway (s)	0.450.68	0.440.55	0.420.59	0.410.38	0.390.43	0.450.54
Target box (s)	2.210.59	1.970.59	1.990.54	2.000.51	1.890.49	1.790.56
Distal (s)	0.200.31	0.370.39	0.240.19	0.140.16	0.220.21	0.280.28
Gaze shift toward box (s)	−2.220.66	−2.060.72	−2.140.55	−1.840.51	−2.010.59	−1.850.50
Gaze shift away from box (s)	0.130.17	−0.030.26	0.050.15	0.110.26	0.020.20	0.010.16
Gaze transfers	1.460.96	1.960.84	1.950.98	1.851.00	1.890.98	2.010.81
Fixation duration (s)	0.560.14	0.540.17	0.510.20	0.530.15	0.530.14	0.500.11
Fixation rate (fix per s)	2.190.69	2.230.59	2.110.67	2.010.65	2.090.52	2.060.60
State-anxiety	1.621.12	2.051.47	2.191.75	1.420.69	1.280.46	1.280.46

#### Gaze Location

As data for gaze location were non-normally distributed, Friedman’s ANOVA’s was utilized to investigate within-participant effects and Mann–Whitney *U*-tests were utilized to investigate between participant effects. A Friedman’s ANOVA showed the allocation of gaze to significantly differ between AOI’s when faced with the target alone, *X*(2) = 54.500, *p* < 0.001, one obstacle, *X*(2) = 52.757, *p* < 0.001, and two obstacles, *X*(2) = 47.197, *p* < 0.001. Follow-up Wilcoxon tests with Bonferroni corrections (α adjusted to 0.0167) showed that for all task difficulties gaze-allocation was significantly greatest for the target-box (*ps* < 0.001), whilst there was no significant difference in gaze allocation between the immediate walkway and distal AOI’s (*ps* < 0.065). A Friedman’s ANOVA also showed distal fixations to significantly change across task difficulties, *X*^2^(2) = 11.123, *p* = 0.004, with Wilcoxon tests with Bonferroni corrections (α adjusted to 0.0167) showing significantly greater distal fixations to occur when faced with either one obstacle, *Mdn* = 7.00%, *z* = -2.855, *p* = 0.004, *r* = -0.4694, or two obstacles, *Mdn* = 7.00%, *z* = -2.812, *p* = 0.005, *r* = -0.4623, compared to the target alone (*Mdn* = 2%). Fixations to the immediate walkway, *X*(2) = 2.279, *p* = 0.320, and to the target box, *X*(2) = 3.748, *p* = 0.153, did not significantly change across task difficulties. Finally, separate Mann-Whitney U tests comparing the spatial allocation of gaze between TD and DCD groups for each AOI, and across each task difficulty, failed to show any significant differences between groups (*ps* > 0.176). These data are presented in Table 2.

#### Gaze Transfer From Target

**FIGURE 3:**
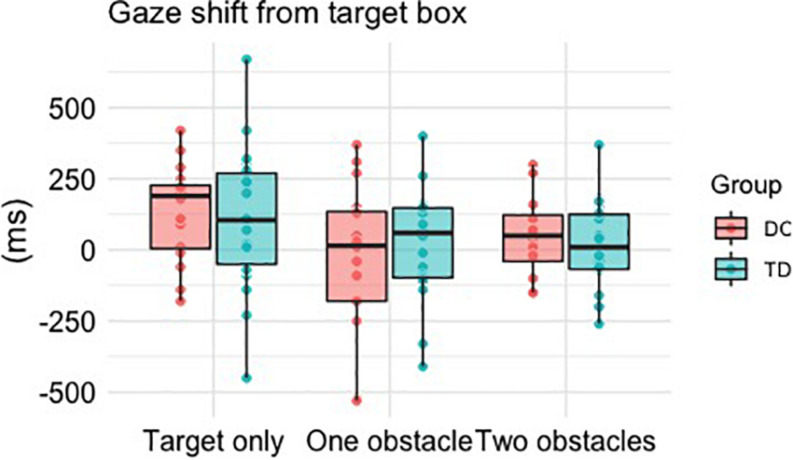
Boxplots and individual mean values for the time taken (ms) to shift gaze away from the target box relative to stepping within it. Positive times reflect gaze to be shifted after foot contact within the box, whereas a negative time reflects an “early” shift of gaze prior to foot contact within the box.

There was a significant main effect of Difficulty, *F*(2, 64) = 8.128, *p* = 0.001, η*_*p*_*^2^ = 0.203, with *post hoc* comparisons revealing significantly earlier transfers of gaze when faced with one obstacle (*M* = -5 ms, *p* = 0.003) or two obstacles (*M* = 30 ms, *p* = 0.031) compared to when faced with the target box alone (*M* = 121 ms). There was no main effect of Group, *F*(1, 32) = 0.004, *p* = 0.948, η*_*p*_*^2^ = 0.000, and no Group × Difficulty interaction, *F*(2, 64) = 0.986, *p* = 0.379, η*_*p*_*^2^ = 0.030 (Figure 3).

### Anxiety

#### State Anxiety

A Friedman’s ANOVA showed anxiety to significantly differ across each instance of measurement, *X*(3) = 12.854, *p* = 0.005, with *post hoc* Bonferroni corrected (α = 0.012) Wilcoxon signed ranks tests revealing significantly higher levels of anxiety at baseline (*M* = 2.06) compared to when faced with the target only (*M* = 1.53, *z* = -2.635, *p* = 0.008, *r* = -0.388) and when faced with one obstacle (*M* = 1.73, *z* = -2.500, *p* = 0.012, *r* = -3.686). Results from separate Mann–Whitney *U*-tests showed no significant differences between groups at any point of measurement (*ps* > 0.075). These data are presented in Table 2.

## Discussion

The present study is the first to quantify foot placement accuracy in children with DCD and to determine the underlying characteristics of gaze and anxiety. Our results show that children with DCD are less accurate than their TD peers when tasked with precisely placing their foot within a floor-based target and are more likely to accidentally contact its edges. The DCD group primarily showed lower foot placement accuracy than the TD group in the anteroposterior plane, which, when considering constant foot placement error (Figure 2), appeared to be a tendency to undershoot the target center (∼1.1 cm). However, the DCD group also displayed greater variable error in both the anteroposterior and mediolateral planes. We therefore provide the first quantifiable evidence that decreases in foot placement accuracy (increased AP error) and precision (increased AP and ML variable error) may be partially responsible for the increased incidence of trips and falls in DCD (Chapman and Hollands, 2007). Interestingly, no differences were observed between groups in the maximal mediolateral CoM velocity during the swing phase into the target box, suggesting that poor foot placement was, in this instance, not underpinned by decreased balance control (Deconinck et al., 2010). In addition to foot placement error, the children with DCD also exhibited significantly greater variability in the length and width of their steps preceding the stepping target. As variability in these gait parameters have previously been observed in adults with DCD (Du et al., 2015) and linked to fall-risk in older adults (Maki, 1997), our findings provide evidence of an inherent deficit in the ability to produce consistent and stable stepping movements in children with DCD.

Contrary to our hypotheses, no differences in gaze behavior were found between groups on any metric reported. Both groups allocated the majority of their gaze toward the target box during the approach toward it, with fixations to the distal pathway minimal, yet increasing when a future obstacle(s) had to be negotiated (∼7% of total gaze). The timing between looking away from the target box and stepping within it was also similar between groups, occurring approximately 120 ms after foot contact when faced with the target alone, and approximately at the instant of foot contact (∼10 ms) following the introduction of an obstacle(s). These similarities may be explained by the fact that our DCD participants did not experience heightened state anxiety pertaining to the completion of our task. Indeed, anxiety was generally low and highly variable in the DCD group, with at least 50% of the cohort reporting the lowest possible levels of anxiety prior to facing each of the three walking tasks. Anxiety was also highest at baseline for both groups, which suggests an anxiety response unrelated to the fear of falling, such as the fear of performing to unfamiliar people in unfamiliar surroundings (i.e., social phobia, see Beidel et al., 1995). Consequently, work is still needed to determine the extent to which gaze and stepping performance might be altered in the presence of heightened anxiety. To achieve this, researchers could explore time-pressure and/or dual-task situations as they have both been shown to induce anxiety (Uemura et al., 2012; Zult et al., 2019) and exacerbate motor difficulties in children with DCD (Wilson and McKenzie, 1998). Alternatively, researchers could explore ecologically valid tasks in which the cost of falling is much greater, such as when walking up and down a staircase. Yet, the similarities in gaze behavior between groups may also be attributable to the predictability of our tasks’ dimensions prior to the target box. As gaze behavior is known to be driven by context complexity and task specificity (Aivar et al., 2005; Miyasike-daSilva et al., 2011), knowledge that the target would consistently be reached on the fourth step for all trials may have reduced between group differences in visual exploration.

Given the similarities in gaze behavior and anxiety between the DCD and TD groups, the results of the present study suggest that difficulties producing precise stepping actions in children with DCD occur independent of anxiety and overt attentional processes related to gaze behavior. As such, fall-risk in children with DCD may be better-explained by general deficits in neuromuscular control and the integration of acquired perceptual information during locomotion. For example, previous research has shown children with DCD exhibit greater variability in their shank and thigh movements during gait, suggesting an inherent difficulty controlling the lower limbs during locomotion (Rosengren et al., 2009). The extent of variability in the shank also appears to be greatest during the stance phase (Rosengren et al., 2009), which might explain increased variability when placing the targeting limb. Furthermore, when walking in dark conditions, children with DCD walk slower and sway more than TD children, suggesting a reduced ability to utilize proprioceptive and vestibular inputs to compensate for visual information and achieve a normal gait pattern (Deconinck et al., 2006b). Children with DCD also display slower and less accurate rapid online control, which is only achieved through the seamless integration of predictive models of movement and feedback mechanisms (Hyde and Wilson, 2011). Deficits in the ability to rapidly integrate information from the visual, vestibular and proprioceptive systems may therefore inhibit the extent to which children with DCD are able to accurately update and correct an ongoing stepping command whilst visually guiding the foot toward a floor-based target (Gentle et al., 2016; Wilmut et al., 2016). However, it is worth acknowledging that, in the absence of any differences in overt attention (spatial location of gaze), differences may still exist in covert attention. Recent evidence has shown increased gait instability to be associated with an internal focus of attention (focusing on one’s own movements) relative to an external focus of attention (focusing on the impact of the movement on the environment; Mak et al., 2019, 2020). Future work should therefore elucidate the covert attentional processes that underpin adaptive gait performance in children with DCD and its relative impact on stepping accuracy.

The results of this study may be limited by several factors. For example, it is important to acknowledge that our sample size is relatively small, and the age range of our participants is relatively heterogenous. Researchers should therefore take care when extrapolating our findings to children with DCD of all ages given evidence that the control of visually guided stepping goes through distinct changes throughout development (Mowbray et al., 2019) and that adaptations to walking on uneven terrain are better distinguished between DCD and TD individuals at childhood as opposed to adolescence (Gentle et al., 2016). Additionally, developmental aspects of emotional self-perception may question the accuracy of our simple self-report measure of state-anxiety (Smith et al., 2006). However, the similarity in gaze behaviors between groups may reinforce a similarity in their experienced anxiety, given the wealth of aforementioned research showing how anxiety can alter visual exploration during locomotor tasks. Regardless, future research would benefit from attempts to objectively capture physiological state-anxiety responses to complement additional measures of self-report. Finally, it should be reiterated that our findings only allow us to comment on the stepping performance of children with DCD in the *absence* of task-related anxiety. It is therefore important for future research to experimentally manipulate anxiety if we are to fully explore its role in fall-risk in children with DCD.

## Conclusion

To conclude, our findings provide the first quantifiable evidence that children with DCD display reduced foot placement accuracy and precision compared to their TD peers. We also provide evidence that these reductions in foot placement accuracy are likely to occur independently of differences in gaze behavior and anxiety, suggesting a general deficit in neuromuscular control and a reduced ability to rapidly integrate perceptual information from the visual, proprioceptive and vestibular systems to guide stepping actions. However, as state anxiety was generally low, more research is needed to explore whether children with DCD may be more susceptible to anxiety-driven maladaptive gaze under more demanding situations.

## Data Availability Statement

The raw data supporting the conclusions of this article will be made available by the authors, without undue reservation, to any qualified researcher.

## Ethics Statement

The studies involving human participants were reviewed and approved by the Liverpool John Moores Ethics Committee. Written informed consent to participate in this study was provided by the participants’ legal guardian/next of kin.

## Author Contributions

MH, RF, and GW acquired the funding and edited the manuscript. MH, RF, GW, and JP designed the study. JP undertaken the data collection, completed the statistical analysis, figure preparation, and the first draft of the manuscript. All authors approved the final version of the manuscript for submission.

## Conflict of Interest

The authors declare that the research was conducted in the absence of any commercial or financial relationships that could be construed as a potential conflict of interest.

## Footnotes

1 www.anxietycanada.com
